# Multidisciplinary inpatient rehabilitation improves the long-term functional status of geriatric hip-fracture patients

**DOI:** 10.1186/s40001-020-00433-2

**Published:** 2020-08-10

**Authors:** Daniel Pfeufer, Christian Kammerlander, Christian Stadler, Tobias Roth, Michael Blauth, Carl Neuerburg, Wolfgang Böcker, Christian Zeckey, Monika Lechleitner, Markus Gosch

**Affiliations:** 1 Department of General, Trauma and Reconstructive Surgery, University Hospital Ludwig-Maximilians-University (LMU) Munich, Munich, Germany; 2 Department for Orthopedics and Traumatology, Kepler University Hospital, Johannes Kepler University Linz, Linz, Austria; 3 Department of Trauma Surgery, Medical University of Innsbruck, Innsbruck, Austria; 4 Depuy Synthes, Luzernstrasse 21, 4528 Zuchwil, Switzerland; 5 Department for Trauma Surgery, Medical University Innsbruck, Innsbruck, Austria; 6 Department for Internal Medicine and Geriatrics, Hospital Hochzirl, Zirl, Austria; 7 Department of Medicine 2/Geriatrics, Paracelsus Medical University, General Hospital Nuremberg, Nuremberg, Germany

**Keywords:** Orthogeriatric care, Hip fracture, Geriatric patients

## Abstract

**Background:**

As the world population ages, the number of hip-related fractures in the elderly is steadily increasing. These fractures generate a major worldwide healthcare problem and frequently lead to deterioration of life quality, mobility and independence in activity of daily life of geriatric patients. At present, many studies have investigated and proved benefits of multidisciplinary orthogeriatric care for elderly hip-fracture patients. Only few studies however, have analyzed treatment concepts for those patients directly following discharge from hospital in specialized rehabilitation centers. The aim of this study was to evaluate effects of a multidisciplinary inpatient rehabilitation on the short- and long-term functional status of geriatric patients who suffered from hip fracture.

**Methods:**

A total of 161 hip-fracture patients aged 80 years and above, or additionally 70 years and above suffering from age-typical multimorbidity were included in this study. Patients who had an initial Barthel Index lower than 30 points were excluded from this study, as most of these patients were not able to attend a therapy at the rehabilitation center due to a poor functional status. The patients were separated into two subgroups dependent on the availability of treatment spots at the rehabilitation center. No other item was used to discriminate between the groups. Group A (*n* = 95) stayed an average of 21 days at an inpatient rehabilitation center that specialized in geriatric patients. Group B (*n* = 66) underwent the standard postoperative treatment and were sent home with further treatment by their general practitioner, nursing staff and physiotherapists. To evaluate the patients’ functional status over the course of time we used the Barthel Index, which was evaluated for every patient on the day of discharge, as well as during checkups after 3, 6 and 12 months.

**Results:**

The average Barthel Index at the day of discharge was 57.79 ± 14.92 points for Group A and 56.82 ± 18.76 points for Group B (*p* = 0.431). After 3 months, the average Barthel Index was 82.43 points for Group A and 73.11 points for group B (*p* = 0.005). In the 6-month checkup Group A’s average Barthel Index was 83.95 points and Group B’s was 74.02 points (*p* = 0.002). After 12 months, patients from Group A had an average Barthel Index of 81.21 while patients from Group B had an average Barthel Index of 69.85 (*p* = 0.005).

**Conclusion:**

The results of this study reveal a significantly better outcome concerning both, short-term and long-term functional status after 3, 6 and 12 months for geriatric hip-fracture patients, who underwent an inpatient treatment in a rehabilitation center following the initial therapy.

## Background

As the world population ages, the number of hip-related fractures in the elderly is steadily increasing, making these fractures a major health care problem all around the world [1, 2]. With growing numbers of fragility fractures, health care provider will face not only demanding medical challenges regarding the treatment of these mostly frail and multimorbid patients, but also a major financial burden [3–6].

Hip-related fractures frequently lead to deterioration regarding geriatric patients’ mobility, life quality and eventually independence in everyday life. Less than half of hip-fracture patients aged 65 years and above regain their prefractural mobility within the first postoperative year [7, 8]. Furthermore, higher rates of morbidity and mortality are well known and proven consequences of fragility fractures in the elderly [9, 10].

At present, many studies have investigated and proved the benefits of multidisciplinary orthogeriatric care for elderly hip-fracture patients [11–13]. Only few studies however, have analyzed treatment concepts for those patients directly following the discharge from hospital in specialized rehabilitation centers [14].

Supplemental to multidisciplinary orthogeriatric care, rehabilitation programs specialized in geriatric patients may further improve the clinical outcome regarding the treatment of fragility fractures, in particular by improving the patients’ functional status and independence in activities of everyday life [15]. The World Health Organization defines the maximization of function and the minimization of limitation in activity and restriction of participation caused from an impairment or disease, as the primary objectives of rehabilitation [16]. Although there are many well implemented rehabilitation concepts for patients with special diseases or impairments such as cardiovascular, pulmonary or neurological disorders, yet there are few established rehabilitation programs specialized in the highly demanding needs of orthogeriatric patients [17–20].

The aim of our study was to evaluate the effects of a multidisciplinary inpatient rehabilitation, not only on the short-term, but also on the long-term functional status of orthogeriatric patients who suffered from hip-related fracture.

## Methods

### Study design and population

This study is a retrospective cohort study (Level of evidence III). The study was conducted at the University Hospital of Innsbruck, which is running a Geriatric Fracture Center specialized in elderly patients suffering from typical age-related problems like cardiovascular diseases or osteoporosis. An orthogeriatric co-management model with trauma surgeons and geriatricians taking care of the patients at the same ward, is implemented at this Geriatric Fracture Center in order to adequately address not only the patient’s fractures, but also age-typical co-morbidities [21]. The initial treatment for all patients took place at this level-I trauma center. The rehabilitation center, which is dedicated to the treatment of geriatric patients, is located at the state hospital of Hochzirl. A team consisting of geriatricians, nurses, physiotherapists, speech therapists, psychologists and social workers provide multidisciplinary care to the rehabilitation center’s geriatric patients, with specialized treatments and exercises on a regular basis in order to restore the patient’s prefractural ability to perform activities of daily living as well as possible. Trauma surgeons are not present at the rehabilitation center permanently, but can be reached out to at all times if there is any need for a surgical consultation. The patients who were not admitted to the specialized clinic were sent home with further treatment by their general practitioner, nursing staff and physiotherapists. All data included in this study were collected during the patients’ clinical treatment and was analyzed retrospectively. As such, there was no study-related change to the standard medical treatment protocol.

Patients older than 80 years with hip fracture treated between August 2009 and November 2011 were included in this study. Furthermore, patients aged 70 to 79 years with hip fracture were included if they additionally suffered from age-typical multimorbidity according to the definition of the German Society of Geriatrics and Gerontology [22]. Further inclusion criteria were a Barthel Index of at least 30 points on the day of discharge from the surgical ward and a complete follow-up over 3, 6 and 12 months.

The study population was retrospectively separated into two subgroups dependent on the availability of free treatment spots at the geriatric rehabilitation center: Group A (*n* = 95) stayed a mean of 21 ± 8 days at the rehabilitation center following the surgical treatment (from those 95 patients, 90 patients (= 95%) were directly discharged to the rehabilitation center, three patients (= 3%) started the rehab within 7 days after the surgical therapy’s end, two patients (= 2%) attended rehab within 19 days). Patients from Group B (*n* = 66) received the standard guideline-conform surgical treatment and were discharged home or to a nursing home. There was no difference regarding the acute phase care (time from admission to hospital to surgery) or regarding the postoperative restrictions (weight bearing) between the two study groups. The mean length of stay at the hospital was 11.61 ± 5.95 days.

All patients were advised to attend checkups after 3, 6 and 12 months at the outpatient clinic.

From a total number of 475 patients with hip-related fractures, 161 patients—134 women (83%) and 27 men (17%)—with a mean age of 82.77 ± 6.51 years met the inclusion criteria mentioned above.

Initially we excluded patients with a Barthel Index lower than 30 points (*n* = 125). From the remaining study population (*n* = 350), 195 patients attended the inpatient rehabilitation at the geriatric rehabilitation center following the surgical treatment (Group A) and 155 patients were discharged home or to a nursing home following the surgical treatment (Group B). In Group A, a total of 21 patients (= 10.77%) died within the follow-up-period of 12 months and 79 patients (= 40.51%) from Group A did not complete the entire follow-up. Altogether we registered a drop-out of 100 patients (= 51.28%) resulting in a population of 95 patients for Group A.

In Group B, a total of 16 patients (= 10.32%) died within the follow-up-period and 73 patients (= 47.10%) from Group B did not complete entire follow-up-plan consisting of checkups after 3, 6 and 12 months. We registered a drop-out of 89 patients (= 57.42%) and 66 remaining patients for Group B.

**Fig. 1:**
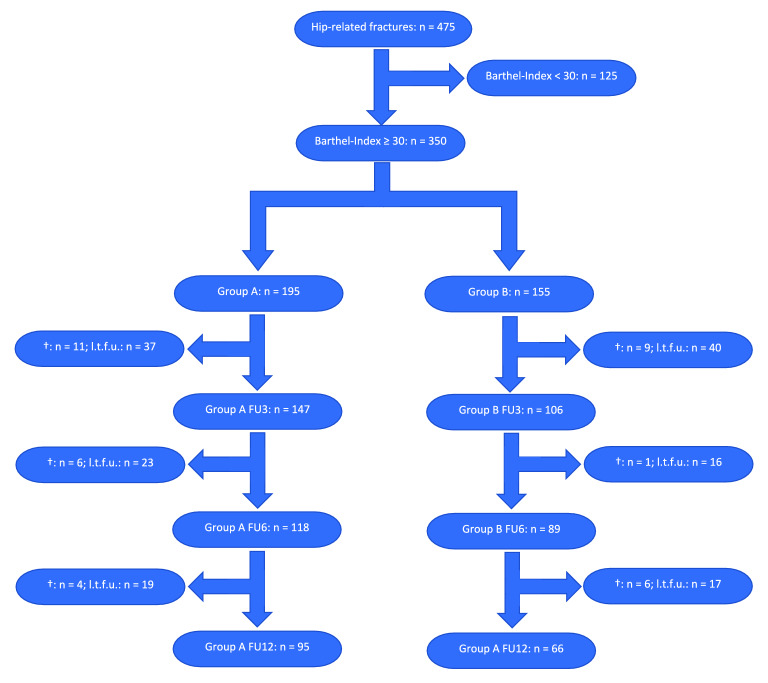
Flowchart showing the detailed numbers of the study’s follow-up and drop-out. (✝: died within the follow-up-period; l.t.f.u.: loss to follow-up; FU3/6/9: follow-up after 3/6/9 months)

Accordingly, we registered an overall drop-out rate of 54.00% (*n* = 189) from those 350 patients who met all of our inclusion criteria resulting in a study population with a total number of 161 patients. For detailed numbers regarding the drop-out please see Fig. 1.

There is a high overall drop-out rate of 54.00% but no significant difference between Group A = 51.28% and Group B = 57.42%.

Patients suffering from one of the following hip-related fractures were included: acetabular fracture, femoral neck fracture, femoral shaft fracture, pubic bone fracture, periprosthetic fracture of the femoral bone and trochanteric fracture (Table 1). The mean follow-up was 372 ± 26 days.

**Table 1:** Baseline patient data within the study population

	Rehabilitation (*n* = 95)	Standard treatment (*n* = 66)	Overall (*n* = 161)	*p* value
Age (years)	82.76 (± 6.16)	82.79 (± 7.04)	82.77 (± 6.51)	0.978
Female patients	81 (85%)	53 (80%)	134 (83%)	0.407
Follow-up (days)	372.77 (± 24)	371 (± 29)	372 (± 26)	0.626

### Data collection

At the time of admission to hospital each patient was surveyed regarding basic information like age, gender, diagnosis, initial treatment, and comorbidities.

On day of discharge we surveyed each patient regarding the current functional status using the Barthel Index, which is used to quantify the patient’s ability to act in activities of daily life, by measuring the occurrence of urinary or fecal incontinence, the ability to perform hair grooming, dressing, feeding, walking, transferring (for example from chair to bed), climbing stairs, using the toilet and bathing independently. Each question is answered by choosing one out of two to four given answers, which are rated in steps of five points with zero points being the minimum for every answer and 10 to 20 points being the maximum depending on the number of given answers [23]. Resulting in an overall score of 100 points at most (= best achievable score) to 0 points at least (= worst achievable score), the Barthel Index is a reliable parameter to measure the independence of patients in daily living [23–25]. During checkups after 3, 6 and 12 months every patient’s basic data and Barthel Index were measured again.

The data collection was carried out by study nurses. The follow-up ended in November 2012.

### Statistics

SPSS version 25.0 (2017) was used for the statistical analysis. To test for normal distribution Kolmogorov–Smirnov test was performed. To analyze non-normally distributed parameters as well as to calculate the significance of the differences between the average scores of the two study groups, non-parametric Mann–Whitney U test was used. *T*-test was performed to analyze differences regarding normally distributed parameters within the two study groups and Chi-square test was used to evaluate nominally scaled data.

As for metric scaled data, arithmetic mean value and the standard deviation were calculated and these two parameters as arithmetic mean value ± standard deviation reported.

To evaluate the effect of an inpatient rehabilitation and certain patient characteristics such as age and gender on the outcome after 12 months, multiple linear regression analysis was conducted. The significance level was defined by *p* = 0.05.

## Results

We included 161 geriatric patients with hip-related-fractures in this study. The mean age was 82.77 ± 6.51 years. 83% (*n* = 134) of the study participants were female while 17% (*n* = 27) were male. The distribution of the baseline patient data within the two study groups is shown in Table 1. The most common types of fractures in the study population were femoral neck fractures (44%; *n* = 71), trochanteric fractures (36%; *n* = 58) or pubic bone fractures (12%; *n* = 20). Other included types of fractures were acetabular fractures, femoral shaft fractures and periprosthetic fractures. The detailed numbers are listed in Table 2. Overall time from admission to hospital to surgery was 22.79 ± 18.25 h. There was no significant difference regarding time to surgery between Group A (22.74 ± 16.68 h) and Group B (22.86 ± 20.91 h) (*p *= 0.974).

**Table 2:** Types of fractures and their frequency within the study population

	Rehabilitation	Standard treatment	Total
Acetabular fracture	4	1	5
Femoral neck fracture	43	28	71
Femoral shaft fracture	2	2	4
Pubic bone fracture	5	15	20
Periprosthetic fracture	3	0	3
Trochanteric fracture	38	20	58
Total	95	66	161

The average Barthel Index on day of discharge was 57.39 ± 16.56 points within the entire study population. After the initial treatment, the study participants either attended a treatment at the rehabilitation center or underwent the standard aftercare—thereby representing our retrospectively created study Groups “A” and “B”.

The mean length of stay at the hospital was 11.61 ± 5.95 days. Patients from Group A stayed an average of 12.05 ± 5.24 days at the hospital while patients from Group B stayed averagely 10.97 ± 6.84 days (*p* = 0.032).

For these two groups, we found the following outcomes: we retrospectively analyzed the Barthel Index on day of discharge for each subgroup separately. Group A had an initial average Barthel Index of 57.79 ± 14.92 points while Group B’s average Barthel Index on day of discharge was 56.82 ± 18.76 points (*p* = 0.431). The patients were either discharged to the geriatric rehabilitation center (Group A), respectively, home or to a nursing home (Group B). From then on, the Barthel Indices were collected separately for each group during the checkups after 3, 6 and 12 months.

**Fig. 2:**
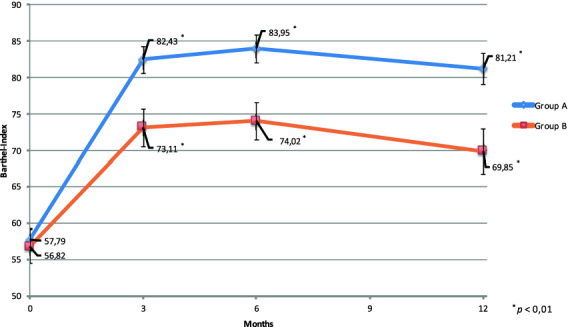
Differences regarding the development of the average Barthel Indices during the follow-up period. Barthel Indices were collected at day of discharge, and after 3, 6 and 12 months

After 3 months, the average Barthel Index of Group A was 82.43 ± 17.95 points and Group B’s was 73.11 ± 21.03 points (*p* = 0,005). In the 6-month checkup, there was a mean of 83.95 ± 18.65 Barthel Index points in Group A while Group B’s mean Barthel Index was 74.02 ± 20.72 points (*p* = 0.002). At the last checkup, 12 months after the initial treatment Group A’s average Barthel Index was 81.21 ± 20.79 points, and Group B’s was 69.85 ± 25.42 points (*p* = 0.005) (Fig. 2).

**Fig. 3:**
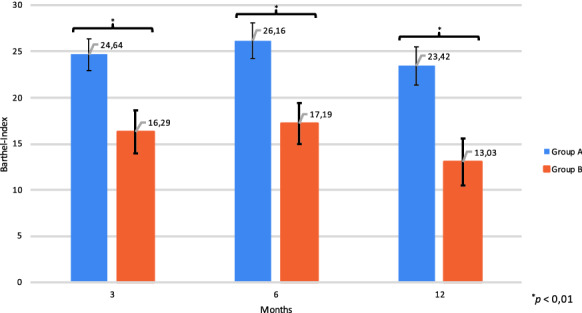
Average difference between the Barthel Indices on day of discharge and the Barthel Indices collected during the follow-up period after 3, 6 and 12 months

The average difference between the initial Barthel Index and the index reported after 3 months was +24.64 ± 16.77 points for Group A and +16.29 ± 18.92 points for Group B (p = 0,008). After 6 months, the difference raised to +26.16 ± 18.77 points in Group A and +17.19 ± 18.06 points in Group B (*p* = 0.004). The improvement regarding the Barthel Index from the day of discharge to the 12-month follow-up was 23.42 ± 20.12 points for Group A and 13.03 ± 20.67 points for Group B (*p* = 0.003) (Fig. 3).

The results of the Mann–Whitney U test are shown in Table 3.

**Table 3:** Results of the Mann–Whitney U test for the non-normally distributed parameters

	Rehabilitation	Standard treatment	Overall	*p* value
Barthel Ind. on day of discharge	57.79 ± 14.92	56.82 ± 18.76	57.39 ± 16.56	0.431
Barthel Ind. after 3 months	82.43 ± 17.95	73.11 ± 21.03	78.61 ± 19.75	0.005
Barthel Ind. after 6 months	83.95 ± 18.65	74.02 ± 20.72	79.88 ± 20.07	0.002
Barthel Ind. after 12 months	81.21 ± 20.79	69.85 ± 25.42	76.55 ± 23.41	0.005
Difference in Barthel Ind. after 3 months	+24.64 ± 16.77	+16.29 ± 18.92	+21.22 ± 18.10	0.008
Difference in Barthel Ind. after 6 months	+26,16 ± 18,77	+17,19 ± 18,06	+22,48 ± 18,95	0.004
Difference in Barthel Ind. after 12 months	+23.42 ± 20.12	+13.03 ± 20.67	+19.16 ± 20.93	0.003

The multiple linear regression analysis evaluates the effects of an inpatient rehabilitation, the patients age, gender and Barthel Index collected at day of discharge on the Barthel Index measured after 12 months. It showed that participation in an inpatient rehabilitation, on average improved the Barthel Index after 12 months by 11.25 points (*p *< 0.001), while females showed an average decline in the Barthel Index by 9.88 points (*p *= 0.016) and every additional year in the patients age by 0.58 points (*p *= 0.019). Additionally, a higher Barthel Index at day of discharge resulted in an averagely improved Barthel Index 12 months later. Detailed results of the multiple linear regression analysis are shown in Table 4.

**Table 4:** Results of the multiple linear regression analysis regarding the effects of an inpatient rehabilitation and certain patient characteristics such as age, gender and Barthel Index at day of discharge on the Barthel Index measured after 12 months

	Regr. coefficient	Standard error	Beta	*T*	*p* value
Rehabilitation	11.246	3.071	0.237	3.662	0.000
Age	− 0.584	0.246	− 0.162	− 2.377	0.019
Gender (female)	− 9.881	4.069	− 0.158	− 2.428	0.016
Barthel Ind. at discharge	0.606	0.096	0.428	6.298	0.000

## Discussion

The aim of this study was to evaluate not only the short, but also the long-term impact of a multidisciplinary inpatient rehabilitation on the functional status of orthogeriatric patients.

Previous studies have investigated and mostly proved the benefits of an inpatient rehabilitation for geriatric hip-fracture patients [14]. However, there are relatively few studies regarding the long-term functional outcome over 12 months—especially when it comes to the use of a reliable parameter for measuring the functional status like the Barthel Index [14].

Our study revealed a significantly better outcome for orthogeriatric patients suffering from hip-related fractures, regarding the functional status over 3 and 6, and even over 12 months, compared to patients who received the standard postsurgical aftercare.

We included 161 patients with hip-related fractures aged older than 80 years, or additionally patients older than 70 years suffering from age-typical multimorbidity—in this study. The two study groups were created retrospectively with the main criteria being the patient’s place of discharge, which depended on the availability of free treatment spots at the rehabilitation center. With a mean age of 82.77 years and the distribution of fractures shown in Table 2, our study population fairly represents the typical frailty-related hip-fracture population [26].

Commonly resulting in a deterioration regarding mobility, life quality and independence in activities of everyday life, fragility fractures are a major threat to orthogeriatric patients [7, 8]. Furthermore, poor functional status is independently associated with higher mortality among geriatric patients [25, 27]. With this study, we intended to evaluate an easily viable approach for enhancing orthogeriatric patients’ functional status by sending them to a specialized rehabilitation center, as the patients’ functional status is—along with the patients’ mobility—one central outcome parameter when it comes to maintaining patients’ quality of life, as well as to reduce the mortality rate of geriatric hip-fracture patients. To measure and evaluate the study participants’ functional outcome, we chose the Barthel Index as it has been proven as a reliable parameter for describing patients’ functional status [23–25].

Early mobilization of geriatric hip-fracture patients is a very important element of the postsurgical aftercare. It is known to reduce the incidence of hospital-related health issues such as delirium or hospital-acquired pneumonia, as well as it is known to lower the in-hospital mortality [28, 29]. Also, early interventions after hip-fracture surgery, such as physiotherapy, are likely to improve the patients’ short-term functional outcome—especially the mobility [30, 31]. A major problem of immobilization is atrophy of muscles. Even short-term muscle disuse of less than 10 days can lead to severe muscle atrophy and in further consequence to the development of sarcopenia [32]. In order to avoid these serious consequences of immobilization, early interventions encouraging the regain of the patients’ prefractural functional status may be a feasible approach for a better long-term outcome, regarding the ability to act independently in activities of daily life among orthogeriatric patients.

Our study’s findings—although several limitations may impair the findings’ reliability—revealed not only a short-term benefit over 3 and 6 months, but also a long-term benefit over 12 months, regarding the functional status of geriatric hip-fracture patients who stayed a mean of 21 days at a rehabilitation center specialized in this challenging patient population, in comparison to patients who received the standard postsurgical aftercare without rehabilitation. In addition, the mean Barthel Index Score of Group A still improved from the 3-month checkup to the 6-month checkup, while the average Barthel Index Score from Group B barely improved (Fig. 2). Due to the complexity and the diversity of every patient’s process of recovery, we are not able to determine the exact reasons for the better outcome of Group A regarding the functional status. It might be possible that early mobilizing interventions directly followed by a multimodal exercise program at the rehabilitation center have given patients from Group A the opportunity to gain and maintain enough muscle strength and coordinative skills, enabling them to perform more independently in activities of daily life than patients from Group B.

## Limitations

There are several limitations to this study. The study’s design is a retrospective uncontrolled single-center setting. The study population consists exclusively of geriatric hip-fracture patients treated at a trauma center specialized on orthogeriatrics. Therefore, our findings relate to this special group of patients and may not be generalizable to other populations of patients or health care settings. Furthermore, we excluded patients with a Barthel Index lower than 30 points from this study. So, we cannot draw conclusions regarding an inpatient rehabilitation’s effects on patients with very low functional status. Also, female patients are over-represented in this study resulting in an under-representation of the male proportion. Therefore, the significance of this study’s findings regarding male patients is limited.

The place of discharge (rehabilitation center, home, nursing home) depended solely on the availability of free treatment spots at the rehabilitation center. Still—due to the retrospective character of this study—we cannot determine for sure if there were certain characteristics to patients, making it more likely getting discharged to the rehabilitation center than characteristics of other patients.

The high drop-out rate of 54% of the initial study population represents another limitation and needs to be considered when interpreting the findings of this study.

Additionally, the use of a single endpoint (Barthel Index) is a big limitation to this study. More measures, for example including gait speed, grip strength or cognitive function would have been additional important parameters when it comes to evaluating the effects of an inpatient geriatric rehabilitation.

## Conclusion

The results of this study reveal a significantly better outcome regarding the long-term functional status of geriatric hip-fracture patients who stay an average of 21 days at a geriatric rehabilitation center, directly following the discharge from hospital. In particular, the study’s findings suggest not only a short-term-benefit from the geriatric-rehabilitation within 3 months after the initial therapy, but also a long-term beneficial outcome after 6 and even 12 months.

From our scope of view, an inpatient rehabilitation as part of the postsurgical aftercare might be a feasible approach to restore the patients prefractural mobility and independence in activities of daily life.

## Data Availability

The datasets used and analyzed during the current study are not available publicly as data were pseudonymized but are available from the corresponding author on reasonable request.
